# Genome-Wide Association Study of Meat Quality Traits in Hanwoo Beef Cattle Using Imputed Whole-Genome Sequence Data

**DOI:** 10.3389/fgene.2019.01235

**Published:** 2019-11-29

**Authors:** Mohammed Bedhane, Julius van der Werf, Cedric Gondro, Naomi Duijvesteijn, Dajeong Lim, Byoungho Park, Mi Na Park, Roh Seung Hee, Samuel Clark

**Affiliations:** ^1^School of Environmental and Rural Science, University of New England, Armidale, Australia; ^2^College of Agriculture & Natural Resources, Michigan State University, East Lansing, MI, United States; ^3^Division of Animal Genomics and Bioinformatics, National Institute of Animal Science, Rural Development Administration, Wanju, South Korea; ^4^Animal Genetic Improvement Division, National Institute of Animal Science, Rural Development Administration, Seonghwan, South Korea

**Keywords:** quantitative trait loci, genome-wide association studies, meat quality, Hanwoo cattle, imputed sequence data

## Abstract

The discovery of single nucleotide polymorphisms (SNP) and the subsequent genotyping of large numbers of animals have enabled large-scale analyses to begin to understand the biological processes that underpin variation in animal populations. In beef cattle, genome-wide association studies using genotype arrays have revealed many quantitative trait loci (QTL) for various production traits such as growth, efficiency and meat quality. Most studies regarding meat quality have focused on marbling, which is a key trait associated with meat eating quality. However, other important traits like meat color, texture and fat color have not commonly been studied. Developments in genome sequencing technologies provide new opportunities to identify regions associated with these traits more precisely. The objective of this study was to estimate variance components and identify significant variants underpinning variation in meat quality traits using imputed whole genome sequence data. Phenotypic and genomic data from 2,110 Hanwoo cattle were used. The estimated heritabilities for the studied traits were 0.01, 0.16, 0.31, and 0.49 for fat color, meat color, meat texture and marbling score, respectively. Marbling score and meat texture were highly correlated. The genome-wide association study revealed 107 significant SNPs located on 14 selected chromosomes (one QTL region per selected chromosome). Four QTL regions were identified on BTA2, 12, 16, and 24 for marbling score and two QTL regions were found for meat texture trait on BTA12 and 29. Similarly, three QTL regions were identified for meat color on BTA2, 14 and 24 and five QTL regions for fat color on BTA7, 10, 12, 16, and 21. Candidate genes were identified for all traits, and their potential influence on the given trait was discussed. The significant SNP will be an important inclusion into commercial genotyping arrays to select new breeding animals more accurately.

## Introduction

The availability of genome-wide single nucleotide polymorphism (SNP) panels has enabled the implementation of genomic prediction in many livestock species. (Goddard and Hayes, 2009; Meuwissen, 2009). Furthermore, many genome-wide association studies (GWAS) have been performed using this SNP information to identify Quantitative Trait Loci (QTL). Results from GWAS, can provide information on the genetic architecture of the quantitative trait and identify potential causative mutations. In comparison to earlier QTL mapping studies using microsatellites, GWAS have resulted in the mapping of QTL with greater precision and have increased the power to detect significant associations. Currently, GWAS are applied to identify candidate genes for many traits in livestock species (McClure et al., 2010; Cole et al., 2011; Gu et al., 2011; Bulik-Sullivan et al., 2015; Hawlader et al., 2017; Strucken et al., 2017). However, significantly associated regions have large confidence intervals, which often result in many candidate genes, which makes it challenging to identify the causative mutation itself. Therefore, fine mapping to identify causal variants in GWAS remains challenging (Teissier et al., 2018). In dairy cattle, despite the fact that a large number of regions have been associated with traits of economic importance at the 50K or high-density SNP panel densities, only very few causative mutations from large QTL have been validated so far (Fortes et al., 2013; Teissier et al., 2018).

Developments in sequencing technologies provide new opportunities to advance the fine mapping of QTLs. Given the relatively high cost of sequencing, there is a trend in which genotyped animals (mostly genotyped for SNP densities between 10 and 700K) are imputed up to whole genome sequence (WGS), and the resulting GWAS has more power and precision to detect significant QTL (Frischknecht et al., 2016; Hawlader et al., 2017). With the application of imputed WGS data in GWAS, all variants including underlying causal variants can be directly genotyped and tested to achieve the simultaneous goal of both discovery and fine mapping (Wang and Chatterjee, 2017). Fine mapping through imputed data is limited if imputation to WGS has low accuracy. The accuracy of imputation can be affected by marker density, effective population size, a sample size of the reference population, genetic distance from the reference population and the phasing accuracy (Browning and Browning, 2009; Zhang and Druet, 2010; Daetwyler et al., 2011; Hayes et al., 2012). These challenges can be overcome with effective experimental design and the application of imputed WGS data. The WGS data in GWAS is a powerful strategy to detect the genetic contributors to complex traits in livestock based on the discovery of new genetic variants that affect the phenotype of an animal, particularly meat quality traits.

Korean cattle (Hanwoo) are categorized into three sub-groups based on coat color: brown, brindle, and black. The Brown Hanwoo is the largest and most common subgroup, and intensive selection has occurred to improve meat quality and quantity traits (Lee et al., 2014; Lim et al., 2016; Sharma et al., 2016). Korean beef consumers prefer Hanwoo beef compared to the imported beef from the US or Australia, mainly due to high intramuscular fat content and excellent flavor of Hanwoo cattle meat (Bulik-Sullivan et al., 2015; Strucken et al., 2017). To meet the consumers’ demands, the selection of the Hanwoo breed has been focused on individuals with high intramuscular fat content (Lee et al., 2014). Few GWAS studies have been reported in Hanwoo cattle for traits like marbling, meat quality and sensory traits (Lee et al., 2010; Kim et al., 2011; Hyeong et al., 2014); however, no studies have examined the meat quality traits such as fat color, meat color and meat texture. Similarly, there is no published study, which uses imputed WGS data for these meat quality traits. The objective of this study was to estimate variance components and to identify significant variants underpinning variation in meat quality traits (marbling score, meat texture, meat color and fat color) using imputed WGS data in Hanwoo cattle.

## Materials and Methods

### Animals and Ethics

All phenotypic and genotypic data were recorded during standard production protocols at Brown Hanwoo Experimental Station, National Institute of Animal Science (NIAS), Rural Development Administration, South Korea. Animal health and welfare issues were followed according to approved guidelines of the Animal Care and Use Committee (NIAS) and the ethics committee approval number was 2015-150. Phenotypic data from 2,110 Hanwoo steers for the traits; marbling score, meat texture, meat color and fat color (meat quality parameters) were used in this study. These individuals were produced from 252 sires and 2,064 dams. All of the data was collected at The Hanwoo Improvement Centre, South Korea during a progeny-testing program between the years 2000 and 2013. The steer progeny (individuals used in this study) were slaughtered at the same age (24 months), and phenotypic measurements were taken on the chilled carcass. The full details of feeding, management practices and traits measurements are reported elsewhere (Bhuiyan et al., 2017; Bhuiyan et al., 2018).

### Carcass Trait Definition

All meat quality traits were recorded using the Korean beef carcass grading system (BCGS). This system was established in 1992, and nationwide implementation occurred in 1999 (Jo et al., 2012). Marbling score was recorded manually by trained technicians using the Beef Marbling Standard (BMS) for grading the carcass. The BMS was originally designed to classify meat based on marbling score with a 2% intramuscular fat (imf) content difference. Based on this threshold, therefore, grade 1 is < 5% imf content, grade 2 is >5%, and ≤ 7%, grade 3 less than 9%, grade 4 less than 11%, grade 5 less than 13%, grade 6 less than 15%, grade 7 less than 17%, grade 8 less than 19%, and grade 9 more than 19%. Similarly, trained technicians graded the other three traits (meat color, fat color and meat texture) manually. Meat color was assessed and graded from very light red (grade 1) to dark red (grade 7). Similarly, the fat color was assessed and graded from polar white (grade 1) to creamy yellow (grade 5). Based on BCGS, the texture of the meat was evaluated on a scale from very fine (grade 1) to coarse (grade 3). Meat quality traits’ mean, minimum (Min), maximum (Max), standard deviation (SD) and coefficient of variation (CV%) is shown in **Table 1**.

**Table 1:** Summary statistics for the four meat quality traits in the 2110 Hanwoo steers.

Traits	Sample size	Min	Mean	SD	Max	CV%
Marbling Score	2,110	1	3.23	1.50	9	46.4
Texture	2,110	1	1.65	0.50	3	30.3
Meat color	2,110	3	4.8	0.55	7	11.5
Fat color	2,110	2	2.98	0.20	5	6.7

### Genomic Data, Imputation and Quality Control

All animals with phenotypic data were genotyped with the 50k SNP Chip (Illumina Bovine SNP50 BeadChip; Illumina, San Diego, CA, version 2). These animals were subsequently imputed to higher densities using a large reference dataset of genotyped individuals. This reference dataset included a total of 4,887 Hanwoo animals genotyped with the 50K SNP chip (Illumina Bovine SNP50 BeadChip; Illumina, San Diego, CA, version 2) and 928 animals genotyped with the 777K SNP chip (Illumina Bovine HD Beadchip, Illumina, San Diego, CA) and 203 reference animals sequenced with an average depth of 25.6X. In the reference dataset, 655 animals were genotyped with both the 50K and 777K chips. Similarly, 140 animals with sequence data were also genotyped with the 50K chip. There were no animals genotyped for both 777K and whole-genome sequence. The imputation process was done in multiple stages. In the first stage, individuals were imputed from the 50K SNP chip up to 777K, followed by an imputation step from 777K up to whole genome sequence level. The phasing and imputation was undertaken, one chromosome at a time, using Eagle version 2.3.2 for phasing and Minimac3 for the imputation (Browning and Browning, 2007; Howie et al., 2012). The accuracy of imputation for WGS was on average 78% for SNPs with a MAF >0.01.The full description of phasing and imputation have been reported previously (Hawlader et al., 2017; Bhuiyan et al., 2018). Only SNPs that were located on the autosomal chromosomes (29 bovine chromosomes) were considered for association analysis. The following quality control thresholds were considered; 1) SNPs that had a genotype call rate less than 90%; 2) SNPs that have less than 1% minor allele frequencies (MAF) and 3) The p-values for Hardy-Weinberg equilibrium (HWE) less than 0.1% were removed. Based on the given thresholds for MAF and HWE, 5,348,000 and 52,000 SNPs were removed respectively. Finally,15,536,497 SNPs passed the quality control thresholds and were used for all analyses.

### Statistical Models and Data Analysis

In the current study, variance components and the resulting heritability for each trait were estimated using bivariate linear mixed effects models implemented in the the GREML module of GCTA (Lee et al., 2012). Prior to fitting these analyses, all possible fixed effects (year and month of birth and group in which the animal was harvested (batch) were tested using ASReml version 4.1 (Gilmour et al., 2015). Consequently, the batch, which represented the contemporary group of the animal, was the only significant fixed effect and was fitted in all subsequent analyses. The description of the contemporary group structure of the experimental animals is shown in **Table 2**. Each of the bivariate models had the general form:

**Table 2:** The description of the contemporary group and population structure of the experimental animals.

Unit	Mean	Min	Max	Total
Contemporary groups	132	11	195	16
Progeny per Sire	8	1	15	
Progeny per Dam	1	1	3	
Number of Sires				252
Number of Dams				2,064
Number of Animals				2,110

(1) y = Xb + Zu + e

Where y is a vector of phenotypes for the traits; b is a vector of fixed effects, u is a vector of additive genetic effects and it was assumed to be distributed as $u|\sigma_{g}^{2}\;∼N\left(0,G\sigma_{g}^{2}\right)\;$ , where, G is the realized GRM calculated from all SNPs inthe imputed WGS data (Yang et al., 2010), and $\sigma_{g}^{2}$ is the genetic variance explained by all SNPs. x and z are the corresponding design matrices that relate the observations to the fixed effects (b) and the random effects (u), e is the vector of residual effects and were assumed to be distributed as $e|\sigma_{e}^{2}\;∼\;N\left(0,\;I\sigma_{e}^{2}\;\right)$ , where $\sigma_{\text{e}}^{2}$ is the residual of the random errors.

The GWAS analysis was undertaken using the MLMA module from GCTA for each trait. The previous model was expanded to include each SNP as a fixed effect and can be represented as:

(2) y = Xb + Wq + Zu + e

Where, q is the vector of allele substitution effects and, W is the matrix of genotype codes (0, 1, 2) for each SNP. As in the previous model, u is a vector of additive genetic effects and it was assumed to be distributed as $u|\sigma_{g}^{2}\;∼N\left(0,G\sigma_{g}^{2}\right)\;$ , where, G is the realized GRM calculated from all remaining SNPs (Yang et al., 2010). All other denotes in the model are described in equation 1. The percentage of genetic variances explained by each significant SNP was calculated according to the following formula:

(3) $$\%Vg=\frac{2pq\alpha^{2}}{\sigma_{g}^{2}}*100$$

Where *p_i_* and *q_i_* are the allele frequencies for the *i_th_* SNP, *α_i_* is the estimated additive effect of the *i_th_* SNP on meat quality traits, $\sigma_{g}^{2}$ is the estimated genetic variance. Fitting a full linear mixed model for each SNP across the genome is computationally challenging. These computational considerations have led to the development of several fast algorithms(Zhang et al., 2010). The Mixed Linear Model Association (MLMA) provides fast implementation of SNP-trait association analysis (Yang et al., 2014). This method has similar implementation procedures with other software tools such as EMMAX, FaST-LMM, and GEMMA. The advantages of the MLMA method include the prevention of false-positive associations due to population or relatedness structure and the method also increase power to detect causal variants by applying a correction that is specific to sample structure.

Often false discovery rate (FDR) of significant SNP is a problem in GWAS. To manage FDR, it is important to check the distribution of p-values. In the current study, the p-values were continuously distributed which showed that the FDR is being controlled. Another problem is often SNP effects are often inflated due to multiple testing of SNP adjacent to each other. A common way to manage multiple testing is using a Bonferroni correction help to decide what SNP are significant or not. However, the Bonferroni multiple testing procedure is commonly perceived as being too stringent in large-scale simultaneous testing situations such as those that arise in the microarray (imputed) data analysis (Diz et al., 2011). Therefore, many researchers advocate alternatives under appropriate circumstances. For instance, the mixed linear model (MLM) increases false negatives while false positives are reduced that means Bonferroni will discard significant observations in the MLM model (Zhang et al., 2010; Bolormaa et al., 2011; Sun et al., 2013). To overcome this challenge, we followed the procedures reported by (Sham and Purcell, 2014; Fadista et al., 2016) to set an alternative p-value threshold. Accordingly, the alternative p-value threshold was calculated as 0.05 divided by the number of independent variants per chromosome then applied to all chromosomes. Independent variants were estimated by removing SNPs based on linkage disequilibrium (LD) (r^2^ = 0.5) using the software PLINK v1.07 (Purcell et al., 2007). The plink procedure considered a window size of 50 SNPs and LD was calculated between each pair of SNPs in the specified window size then remove one of a pair of SNPs if the LD was greater than 0.5. Therefore, in the current analysis, we applied, Bonferroni corrected genome-wide test and an alternative threshold of p-value (p < 1x 10^−6^) that is less stringent than the Bonferroni threshold to avoid false negative and to control false positive due to multiple testing.

The online database (Ensembl) under Cow (*Bos taurus*) UMD3.1 assembly was used for the annotation of significant SNPs. Finally, LocusZoom standalone version (Pruim et al., 2010) was used to visualize regional association and LD of SNPs.

## Results

### Variance Component Analysis

The estimated heritabilities for marbling score and meat texture were 0.49 ± 0.05 and 0.31 ± 0.04, respectively. The heritabilities for meat and fat color traits were 0.16 ± 0.04 and 0.01 ± 0.03, respectively. The genetic correlation of marbling score with meat and fat color were low and negative (−0.14 ± 0.08 and −0.13 ± 0.10), respectively. Conversely, the genetic correlation between marbling score and meat texture was high (−0.97 ± 0.02) and the genetic correlation between meat and fat color was moderate and positive (0.43 ± 0.15). Meat color showed moderate genetic correlation (0.66 ± 0.13) with meat texture and, fat color also showed moderate genetic correlations with meat texture (0.52 ± 0.15). Estimated variance components are reported in **Table 3**.

**Table 3:** Variance components, heritabilities, genetic and phenotypic correlations for marbling score (ms), texture (tex), meat color (mc), and fat color (fc) for Korean Hanwoo cattle.

Parameters	Traits			
	Marbling score (ms)	Texture (tex)	Meat color (mc)	Fat color (fc)
Genetic variance	1.05(0.13)	0.07 (0.01)	0.04 (0.01)	0.0003(0.001)
Residual variance	1.08(0.10)	0.15 (0.01)	0.212 (0.01)	0.04 (0.001)
Phenotypic variance	2.13 (0.07)	0.22 (0.01)	0.25 (0.01)	0.04 (0.001)
Heritability	0.49 (0.05)	0.31 (0.04)	0.16 (0.04)	0.010 (0.03)
Genetic correlation (ms)	–	−0.97 (0.017)	−0.14 (0.085)	−0.13 (0.13)
Genetic correlation (tex)	–	–	0.66 (0.13)	0.52 (0.15)
Genetic correlation (mc)	–	–	–	0.43 (0.15)

### GWAS Analysis

**Figure 1:**
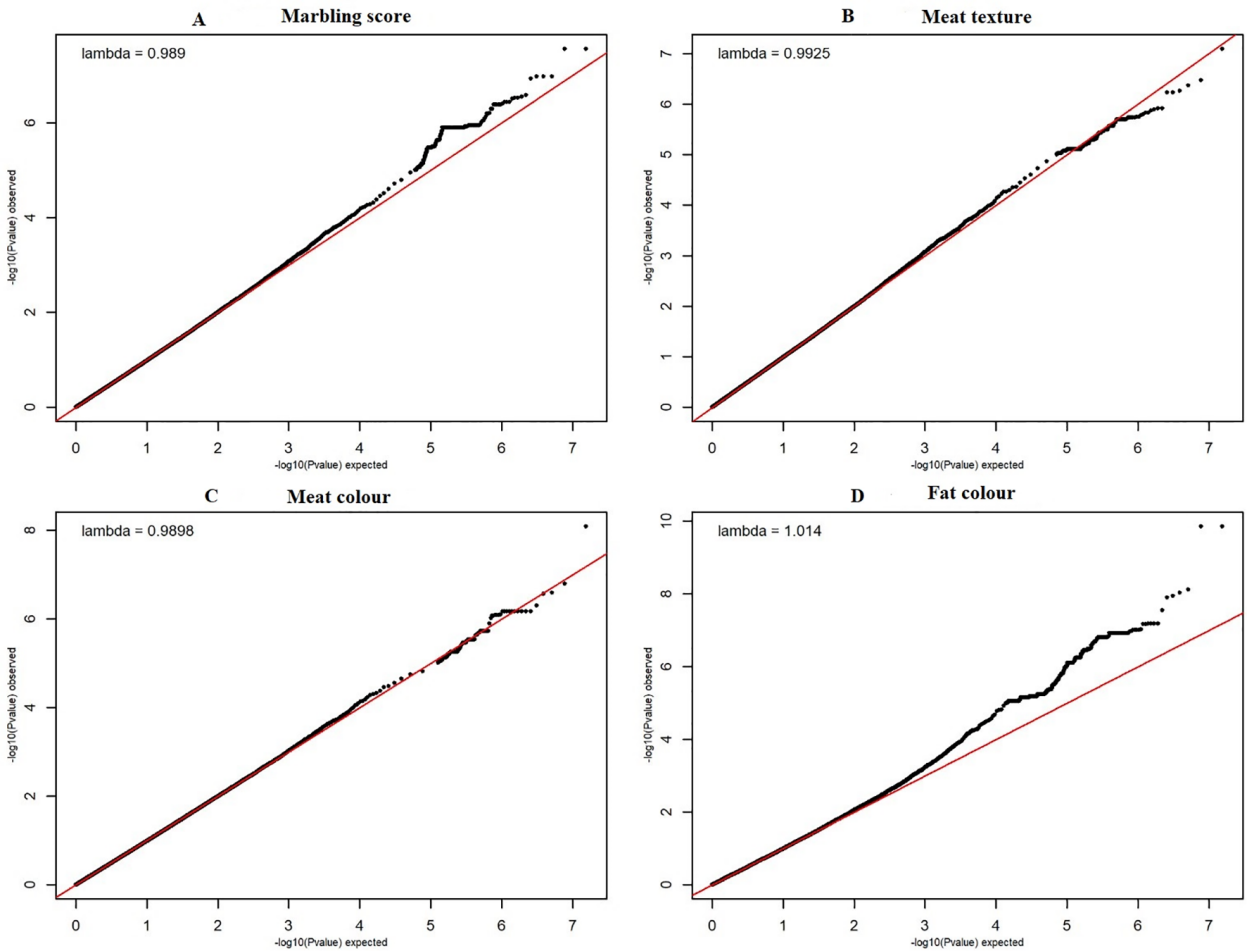
The quantile-quantile (QQ) plots for the studied meat quality traits. The figure showed quantile–quantile plot for each meat quality traits with genomic inflation control (lambda) value. The red line represents the 95% concentration band under the null hypothesis of no association among traits and SNPs. The black dots represent the P-values of the entire study. The panels **(A**–**D)** have shown the QQ plots for marbling score, texture, meat and fat color traits respectively.

**Figure 2:**
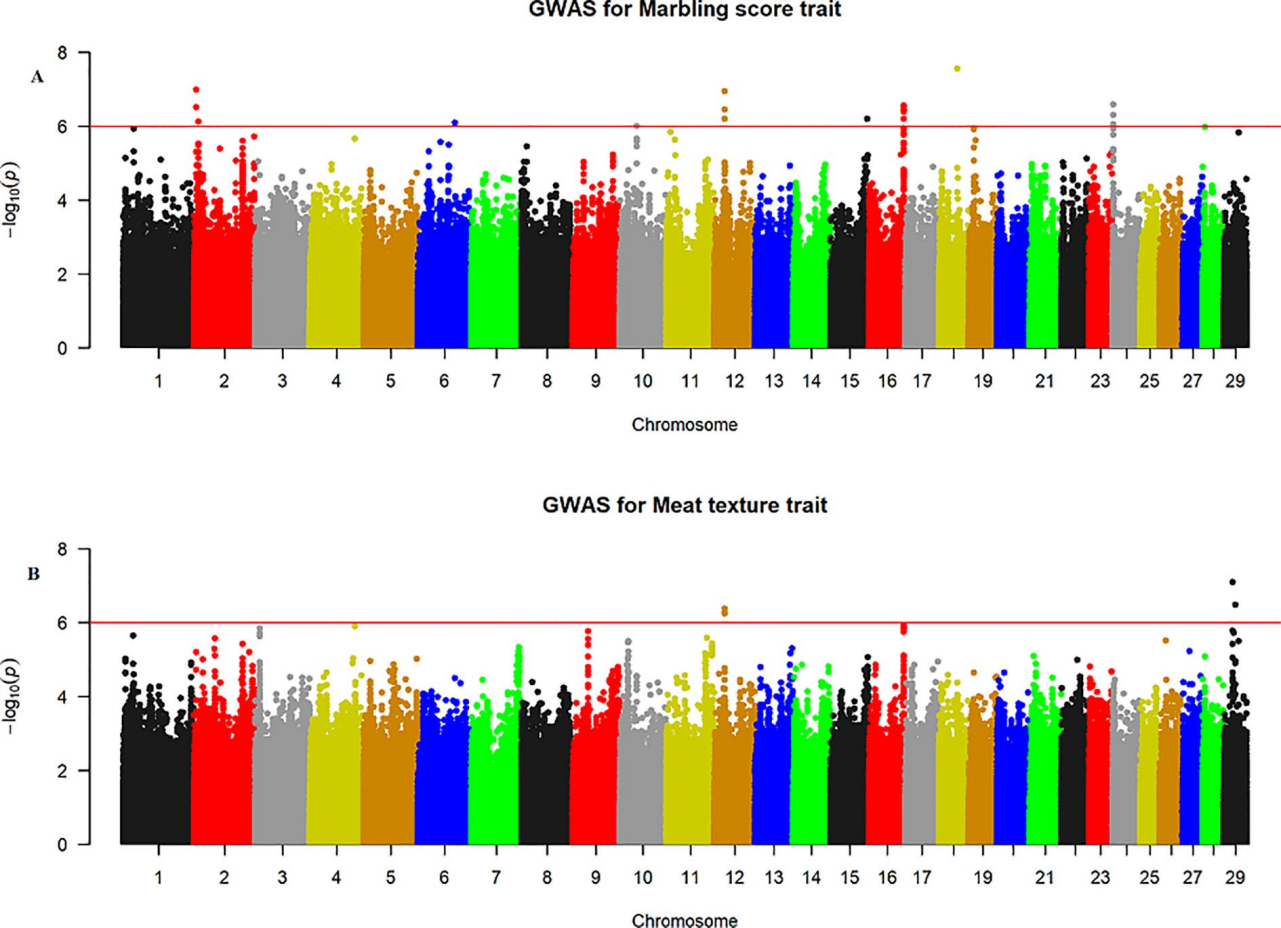
Manhattan plots of WGS for marbling score and meat texture with significance thresholds indicated at −log_10_P >1×10^−6^. Panel **(A)** and **(B)** show that the chromosome regions that were associated with marbling score and meat texture traits respectively, using ∼15 million imputed sequence SNPs.

**Figure 3:**
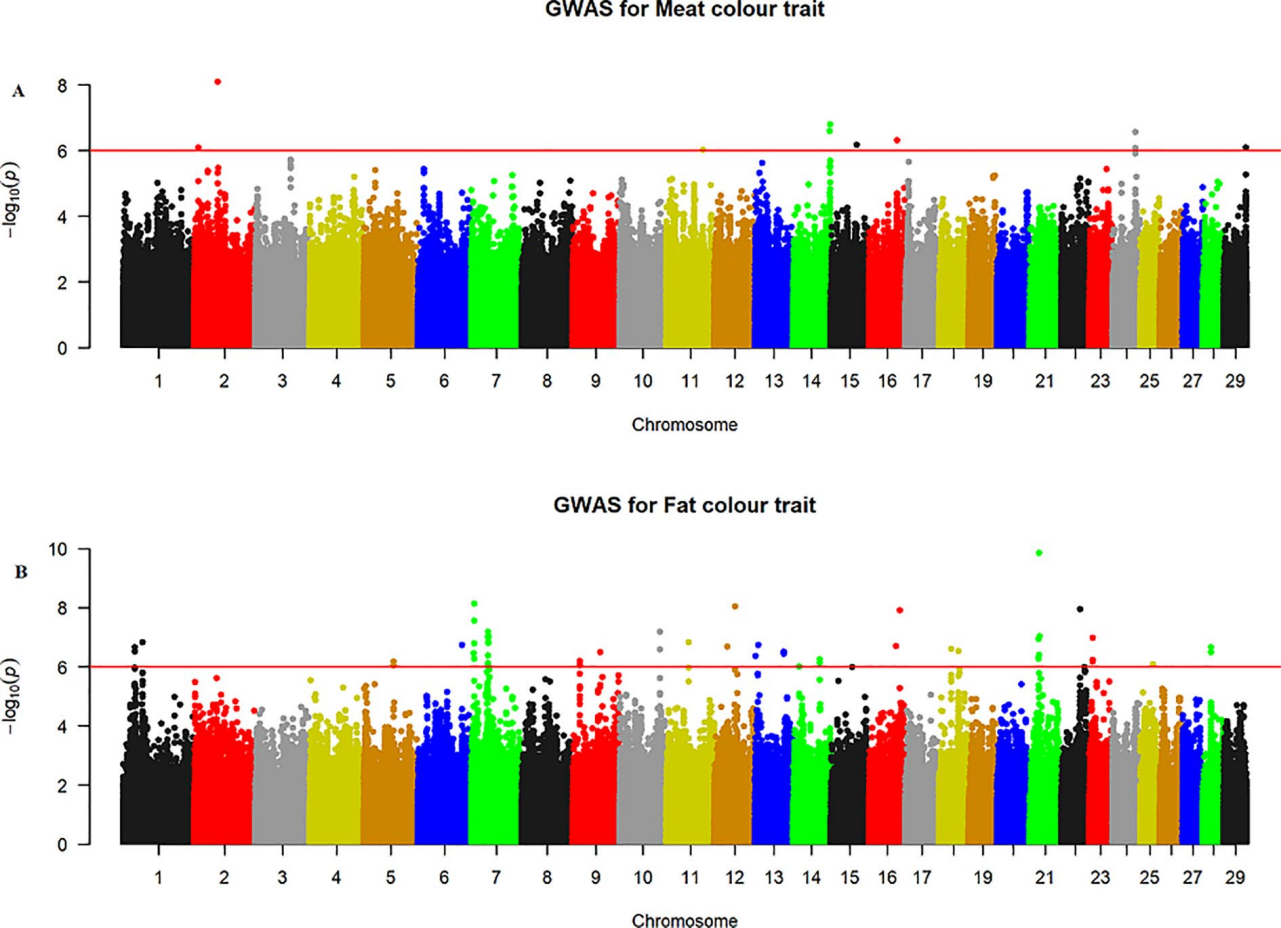
Manhattan plots of WGS for meat color and fat color with significance thresholds indicated at −log_10_P >1×10^−6^. Panel **(A)** and **(B)** show that the chromosome regions that were associated with meat and fat colour traits respectively, using ∼15 million imputed sequence SNPs

The quantile–quantile (QQ) plot for each trait showed that the model (MLM) fitted the data well. Genomic inflation factor (lambda value) obtained from the QQ plot indicated low bias and ranged between 1 and 1.04 for all studied traits and shown in **Figure 1**. In total 107 SNPs passed the alternative significance (p < 1×10^−6^) p-value threshold, located on 14 selected chromosomes (one region per selected chromosome). For each trait, only the most significant regions were selected for further downstream analysis. For the trait of marbling score, the GWAS distinguished four significant regions across four chromosomes comprising of 31 significant SNPs that passed the alternative p-value threshold (p < 1×10^−6^). The additive genetic variance explained by each significant SNP varied from 2 to 3.6%. The GWAS for meat texture identified two significant regions which comprised of 13 SNP, on two chromosomes, that passed the alternative p-value threshold (p < 1×10^−6^). The additive genetic variance explained by each significant SNP varied from 3 to 3.6%. The three most significant regions (p < 1×10^−6^) were considered for meat color. These regions included 22 SNPs on three separate chromosomes, and each SNP explained 3 to 6% of the additive genetic variance. Five significant regions comprising of 41 SNPs across five chromosomes were identified for fat color (p < 1×10^−6^), among the 41 SNPs, five SNPs reached the Bonferroni threshold. The additive genetic variance for fat color explained by each significant SNP varied from 3 to 9%. Manhattan plots illustrating the results from the GWAS for marbling score (panel, A) and meat texture (panel, B) are shown in **Figure 2**. Manhattan plots for meat color (panel, A) and fact color (panel, B) are shown in **Figure 3**.

#### Marbling Score

**Figure 4:**
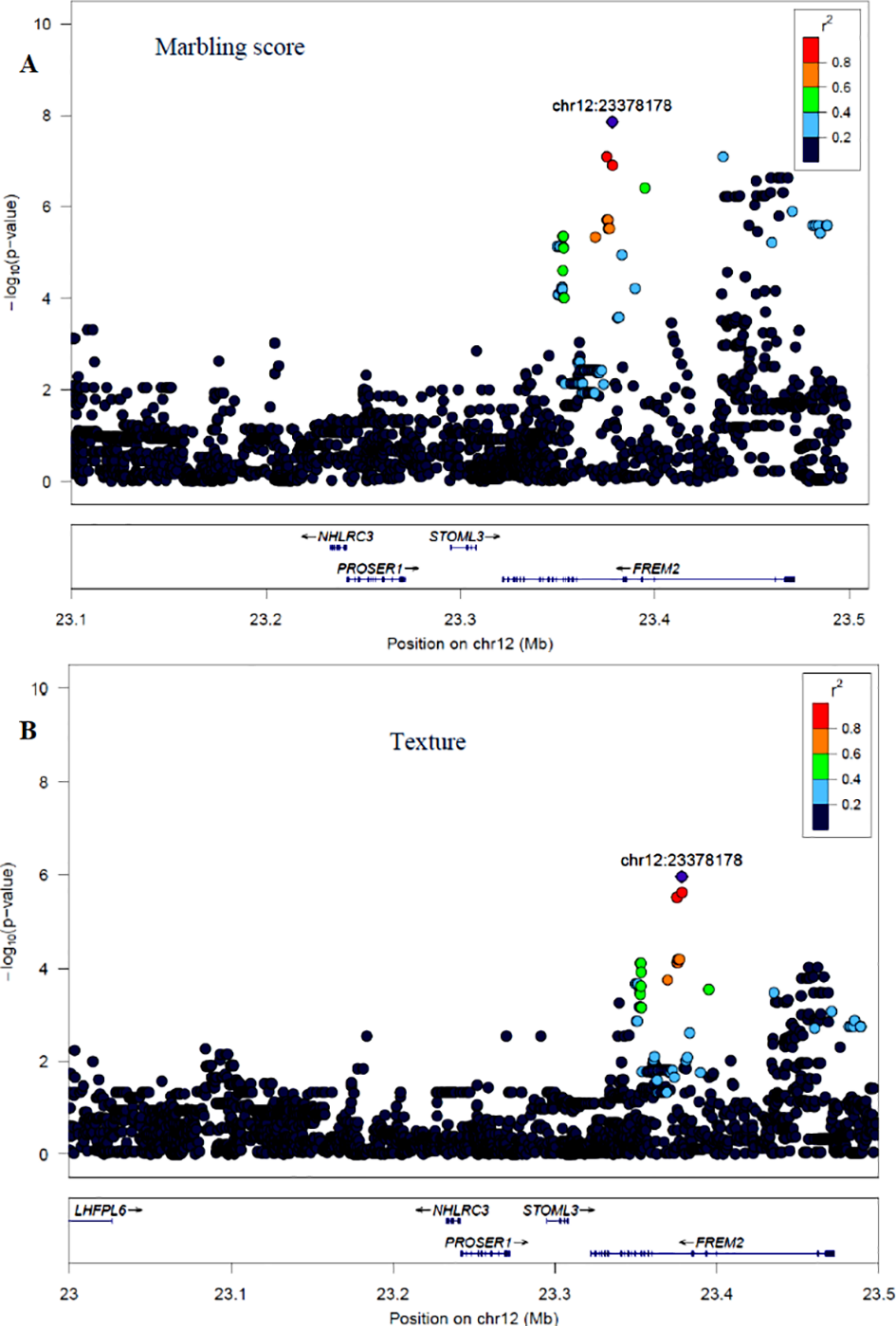
LocusZoom plots for the top significant SNP that was associated with marbling score (panel, **A)** and meat texture (panel, **B)** within 0.5 Mb of a genomic region on BTA12. The bottom panel of a LocusZoom plot shows the name and location of genes in the UCSC Genome Browser. Positions of exons are displayed, and the transcribed strand is indicated with an arrow. Gene names are automatically spaced relative to one another to avoid overlap.

The GWAS for marbling score identified four significant regions (**Figure 2A**). Each region comprised 5, 4, 10, and 12 SNPs on BTA2, 12, 16, and 24, respectively. The significant SNPs on BTA2 were located between 4.75–4.78 Mb and the most significant (p = 1.06×10^−7^) SNP (rs43287038) explained 3.4% of the genetic variance. Among the five significant SNPs located at 23.38 Mb on BTA12, the most significant SNP was rs208621284 with the p-value of 1.17×10^−7^ and explained 3.7% of the genetic variance. These five SNPs were in high LD with each other (**Figure 4A**). Similarly, 10 significant SNPs were located on BTA16 and spanned at 78.5–78.6 Mb. The most significant SNP on BTA16 was rs133022670 with the p-value of 2.8×10^−7^, and 3.4% of the genetic variance was explained by this SNP. Furthermore, 12 significant SNPs were located on BTA24 and spanned between 2.2–2.3 Mb. The most significant SNP on BTA24 was rs134591476 with the p-value of 2.6×10^−7^, and 3.13% of the genetic variance was explained by this SNP. According to Ensembl’s variant effect predictor (VEP) database, these significant SNPs are annotated as intron variants (56%), and 44% of those SNPs are annotated as intergenic variants. Intergenic regions were defined as regions more than 5 Kb distant from genes whereas regulatory regions were defined as regions located 5 Kb upstream and 5 Kb downstream of genes. Detail description of selected top significant SNPs including effect size of each SNP and adjacent genes to the significant SNPs on 14 selected chromosomes (**Supplementary Material Table 1**). The most significant SNP (chr12:23378178/rs208621284) associated with marbling score was in high LD with the next five significant SNPs. Among the five top significant SNPs, rs210129449 (chr12:23375305) SNP showed the highest LD with top significant SNP with the value of r^2^ 0.83. Within 0.5 Mb genomic region, four candidate genes (*NHLRC3*, *PROSER1*, *STOML3*, and *FREM2*) were identified close to the most significant SNP that was associated with marbling score and meat texture, particularly the top significant SNP (rs208621284) was found in the transcribed strand region of the *FREM2* gene. LocusZoom plots for the top significant SNPs that were associated with marbling score (panel, A) and texture (panel, B) are shown in **Figure 4**.

#### Meat Texture

The GWAS for meat texture identified significant regions on BTA12 and BTA29 (**Figure 2B**). Based on the alternative p-value threshold (P < 1×10^−6^) four SNPs on BTA12 and seven SNPs on BTA29 were detected. The four most significant (p = 5.81×10^−7^) SNPs (rs210266948, rs208621284, rs210129449, and rs208265955) were located at 23.4 Mb on BTA12. This peak consisted of the same QTL region that was identified for marbling score. Interestingly, another significant region was identified on BTA29, with the most significant SNP (BTA29:19924266 with a p-value of 8.04×10^−7^) located at 19.9 Mb and explained 4.8% of the total genetic variance. All significant SNPs from both chromosomes (BTA12, 29) were annotated as 45% intron, 36% intergenic, and 18% downstream gene using VEP in Ensembl database. Detail description of selected top significant SNPs including effect size of each SNP and adjacent genes to significant SNPs on BTA12 and 29 (**Supplementary Material Table 1**).

#### Meat Color

**Figure 5:**
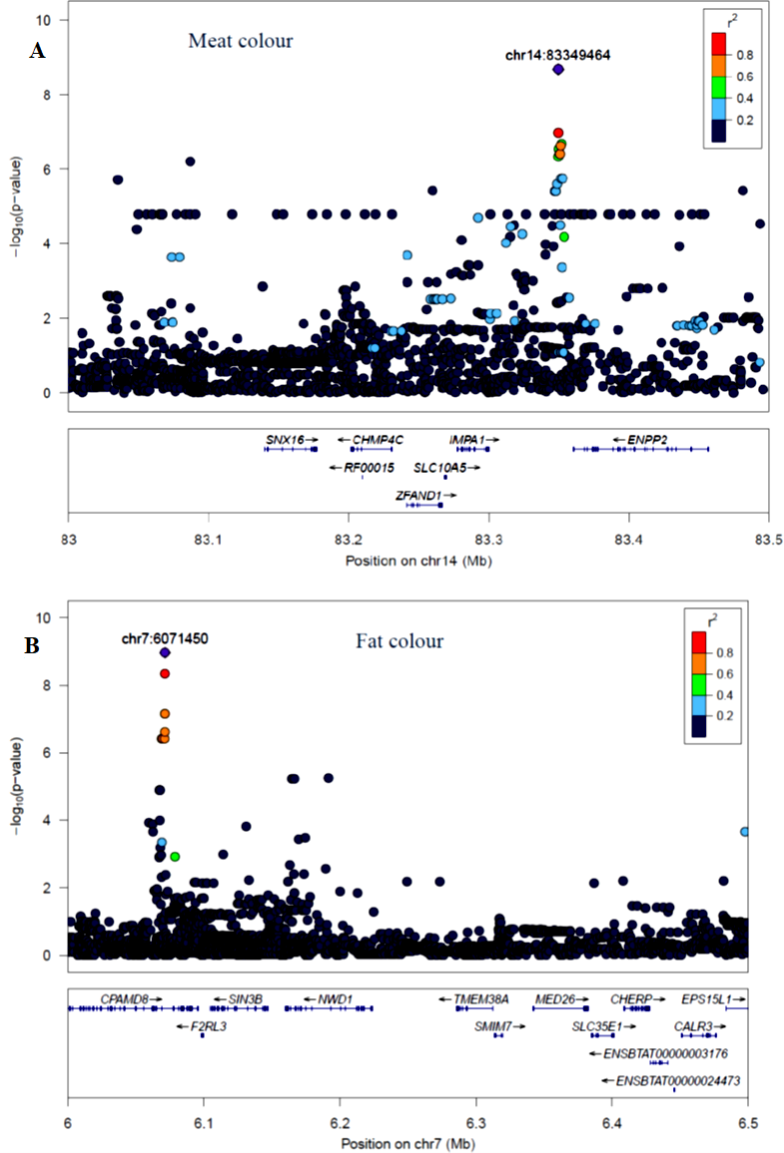
LocusZoom plots for top significant SNP associated with meat color (panel, **A**) on BTA14 and fat color (panel, **B**) on BTA7 within 0.5 Mb of a genomic region. The bottom panel of a LocusZoom plot shows the name and location of genes in the UCSC Genome Browser. Positions of exons are displayed, and the transcribed strand is indicated with an arrow. Gene names are automatically spaced relative to one another to avoid overlap.

The GWAS identified three major regions strongly associated with meat color on chromosome 2, 14, and 24 (**Figure 3A**). On each chromosome, there were nine (BTA2), eight (BTA14), and five (BTA24) significant SNPs identified. The most significant (p = 8.14×10^−7^) SNP found on BTA2 was rs210985952 and located at 9.57 Mb. Eight significant SNPs were located on BTA14 at the similar genomic position of 83.35 Mb, and the most significant SNP (p = 1.6×10^−7^) on this chromosome was rs137372673, and it explained 4.5% of the genetic variance. Similarly, eight significant SNPs spanned between 51–53 Mb on BTA24 and the most significant SNP (p = 8.5×10^−7^) on BTA24 was rs110365059, and it explained 4.3% of the genetic variance. All SNPs that were associated with meat color located on BTA2 were annotated as an intron variant in VEP/Ensembl database while all SNPs that were located on BTA14 and 24 were annotated as intergenic variants. Detail description of selected top significant SNPs including effect size of each SNP and candidate genes that were associated with significant SNPs on chromosome 2, 14, and 24 (**Supplementary Material Table 1**). The top significant SNP (chr14:83349464/rs137372673) associated with meat color was in high LD with the other four SNPs on BTA14. Among these SNPs, chr14:83349474/rs133028626 SNP showed the highest LD with the top significant SNP with the value of r^2^ 0.955. Within this genomic region (0.5 Mb), seven candidate genes (*SNX16*, *CHMP4C*, *RF00015*, *ZFAND1*, *SLC10A5*, *IMPA1*, and *ENPP2*) were identified, particularly the putative variant (rs137372673) was found in the transcribed strand region of the *ENPP2* gene. LocusZoom plots for the top significant SNPs that were associated with meat color (panel, A) and fat color (panel, B) are shown in **Figure 5**.

#### Fat Color

The GWAS for fat color detected five significant regions on five chromosomes (**Figure 3B**). Ten significant SNPs were identified on BTA7, three on BTA10, two on BTA12, four on BTA16 and 22 on BTA21. The two most significant SNPs (rs209994670 and rs382047302 with a p-value of 1.4×10^−10^) were located on BTA21 at 21.77 Mb. The other 20 significantly associated SNPs were located on BTA21 spanning a region of 21.38–21.77 Mb. Apart from BTA21, the next most significant (p = 7.45×10^−9^) SNP (rs210647091) was located on BTA7 at the genome position of 6.1 Mb. The three significant (p < 1.8×10^−7^) SNPs that were found on BTA10 were located at 89.6 Mb. On BTA16, the most significant (p = 1.24×10^−8^) SNP (BTA16:69913004) was located at genome position of 69.91 Mb. Similarly, the most significant SNPs (BTA12:46590012 with the p-value of 9×10^−9^) was located at 46.59 Mb. There were also a number of single SNP across multiple chromosomes that showed association with fat color; however, these were not considered for further QTL region analysis. According to VEP (Ensembl), all significant SNPs that were associated with fat color were categorized as 42% intergenic, 31% upstream gene, 23% intron, and 4% downstream gene variants. Detail description of selected significant SNPs including effect size of each SNP and adjacent genes to the significant SNPs on chromosome 7, 10, 12, 16, and 21(**Supplementary Material Table 1**). The top significant SNP (chr7:6071450/rs210647091) associated with fat color was in high LD with seven other SNPs on BTA7. Among the seven SNPs, chr7:6071393/rs208014556 SNP showed the highest LD with the top significant SNP with the value of r^2^ 0.94. Within this genomic region (0.5 Mb), four candidate genes (*CPAMD8, F2RL3, SIN3B and NWD1*) were identified, particularly the putative variant (rs210647091) was located within the *CPAMD8* gene region (**Figure 5B**).

## Discussion

### Phenotypic Variation and Variance Component Analysis

This is the first study on Hanwoo beef cattle that has estimated the genetic parameters for meat texture, fat color and meat color using WGS data. A previous study (Lee et al., 2013) on Hanwoo cattle using 50K genotype information has shown that the heritability of marbling score is high (0.4 to 0.57) which is in agreement with the result (0.49) obtained from the current study. These results were substantially higher than previously estimated based on pedigree information (0.28) using phenotypic data collected from the larger Hanwoo beef cattle industry (Do et al., 2016a). The other examined traits were also heritable with the estimated heritability of (0.01) for fat color, (0.16) for meat color and (0.31) for meat texture. This is again in agreement to results from industry-based data where heritability estimates for these traits were reported as 0.06 for fat color (Do et al., 2016a). However, Do et al. (2016a) reported lower heritability (0.14) for meat texture and 0.06 for meat color compared to the result obtained from the current study that was 0.31 for texture and 0.16 for meat color. While this is one of the first study to examine the aforementioned traits in Hanwoo beef cattle, the traits have been examined in other beef cattle populations around the world (Crews and Kemp, 2001; Riley et al., 2002; Yoon et al., 2002; MacNeil et al., 2010). MacNeil et al. (2010) reported high heritability estimates of 0.48 for marbling score in Angus beef cattle. High heritability estimates for marbling have been further validated in, crossbred F1 Wagyu Limousin beef cattle (0.55) (Crews and Kemp, 2001), and Brahman (0.44) (Riley et al., 2002). More moderate heritability estimates have been obtained for other traits like meat texture. In a recent study on Japanese black cattle, Inoue et al. (2017) reported 0.40 heritability for meat texture which is higher than the currently estimated value (0.31) in Hanwoo beef cattle. In the current study, the heritability for meat color was lower (0.16) and lower than estimated by Aass (1996) 0.27 and (Renand, 1988) 0.26, who reported heritability for meat reflectance (color) in European dual-purpose (Norwegian Red) cattle. The estimated heritability for fat color ranges from zero to 0.3 in other beef cattle populations (Yoon et al., 2002; Inoue et al., 2017) which is in agreement with the current results.

In the current study, the genetic correlation between marbling score and meat texture was high (−0.97), and this estimate is very similar to the one estimated by Do et al. (2016b) (−0.96). The high genetic correlation between marbling score and meat texture suggests that the traits are highly dependent. Given this strong relationship and the fact that the marbling score has more genetic variation, it is the easiest trait to use to select for high-quality meat. In addition to this, selection based on marbling will also result in simultaneous genetic improvement in meat texture. The genetic correlation between meat color and fat color was moderate (0.43) which is in agreement with (Do et al., 2016b) who estimated 0.60 in Hanwoo cattle. Similarly, in the current study, the correlation of meat texture with meat and fat color was 0.66 and 0.52, respectively. These estimates were similar with the previously reported by Do et al. (2016b) (0.54) between meat texture and meat color and 0.57 for meat texture with fat color). Given this, our results suggest that limited opportunities exist to exploit marbling as an indirect means of assessing the color traits.

A major reason for the slight differences between the results obtained in the current study and those previously reported are that the data used in this study was from a controlled progeny test whereas data in another study (Yoon et al., 2002) was from broad-scale industry collection. This industry data varied more in age and management prior to harvest. Other possible reasons for the differences include; type of genetic information (pedigree or genomic), sample size, differences between meat grading technicians (due to its subjectiveness) and measuring techniques (measuring devices versus subjective scoring).

### GWAS for Marbling Score and Meat Texture Traits

The GWAS detected 31 significant SNPs associated with marbling score. Significant regions on BTA2 have previously been reported by several authors in many beef cattle populations (Alexander et al., 2007; Abe et al., 2008; McClure et al., 2010; Mateescu et al., 2017). Alexander et al. (2007) identified a possible QTL in an F2 population from a cross between Japanese Black and Limousin on chromosome 2 that spanned at 4.2–5.3 Mb. A similar region was noted by Abe et al. (2008) who reported a possible QTL spanning 4.3–4.5 Mb (again in a Japanese Black crossed Limousin population). Given that this region has been found in a variety of cattle populations, it is likely that a true QTL associated with marbling score is located on BTA2. Among the identified candidate genes for this region, *SFT2D3* gene was the nearest gene to the most significant SNP. According to Gupta et al. (2015), *SFT2D3* gene was associated with the synthesis of lipoproteins. Lipoproteins are a core component of fat and cholesterol cells and have been linked with lipid cell membrane development. This provides further evidence that a QTL for marbling is located in this region.

Apart from BTA2, McClure et al. (2010) identified a potential QTL on BTA12 spanned at 6.9–11.0 Mb in Angus cattle; however, in the current study, we identified a QTL region on BTA12 at a genomic position of 23 Mb in Hanwoo beef cattle. The top significant SNP (rs208621284) associated with marbling score trait showed high LD (0.83) with the five adjacent SNPs, and these SNPs are located in the transcribed strand region of the *FREM2* gene (**Figure 4A**). However, no prior GWAS evidence exists revealing this gene is associated with marbling score in cattle; however, the FREM2 gene has been shown to be involved in adipogenesis in human (McGregor et al., 2003).

Potential QTL regions associated with marbling score in beef cattle have been previously identified on BTA16 by Mateescu et al. (2017). These QTL regions coincide with the region identified in the current study on BTA16. A potential candidate gene (bta-*mir-2284n*) is located close to the most significant SNP. According to Zhang et al. (2016), the bta-mir-2284n gene is associated with the development of bovine mammary epithelial cells. Mammary epithelial cells are often linked to adipose tissue; however, the role of this gene with marbling in the group of Hanwoo bulls used in this study is unclear.

Similar to BTA 16, McClure et al. (2010) also revealed a potential QTL that was located at 1.8–5.5 Mb on BTA24 in Angus cattle, which is in agreement with a QTL that was identified in the current study. The *GALR1* gene was a candidate gene located close to the most significant SNP on BTA24 and it is responsible for neuropeptide and peptide hormone bindings (Jurkowski et al., 2013). In addition to this, the *GALR1* gene has been linked to the synthesis of bioactive lipids (Contos et al., 2002). A bioactive lipid is a lipid for which changes in lipid levels result in functional consequences. In a recent study by Bermingham et al. (2018), many bioactive lipids (especially phospholipids and fatty acids) were associated with marbling in Wagyu dairy cross beef cattle. All peaks identified as significant regions have been linked to genes that have clear links to fat development in humans. This information combined, with the statistical analysis, indicates that these are in fact QTL regions important for the development of marbling in Hanwoo beef cattle. Similar regions were identified for meat texture and marbling on chromosome 12 which is not surprising given the high relationship between the two traits. Surprisingly, one additional potential QTL was identified on BTA29 not previously reported for meat texture. However, Curi et al. (2009) identified a potential QTL on BTA29 that spanned at 35.0–35.1 Mb associated with myofibrillar fragmentation index trait in beef cattle. Myofibrillar fragmentation potentially affects meat quality traits, and it is highly associated with meat texture and tenderness (Li et al., 2014). As previously noted, the region on chromosome 12 and therefore the identified candidate gene (*FREM2*) (**Figure 4A**) was shown to have a potential impact on marbling score. There is no evidence to show that this gene has an impact on meat texture. Additional candidate gene (*ANO5*) was identified on BTA29 that was potentially associated with meat texture and the gene (*ANO5*) was known for its involvement in the formation of amino acids (Sarkozy et al., 2013).

The most significant SNP (rs208621284) associated with meat texture showed high LD with the adjacent significant SNPs (**Figure 4B**). The extent of LD in Hanwoo cattle was lower compared to European beef and dairy cattle. According to (Lee et al., 2011), the extent of LD (r^2^) in Hanwoo was 0.23 for pairwise distances of less than 25 kb, and LD dropped to 0.1 for 40 to 60 kb. These estimates of LD concur with LD estimates from the Hanwoo cattle used in the current study (result not shown). Similarly, Sharma et al. (2016) reported low LD (r^2^ = 0.1) and high heterozygosity (0.4) in brown Hanwoo cattle compared to other Asian cattle. Furthermore, Sharma et al. (2016) suggested that the recent selection of brown Hanwoo for meat quality is the reason behind the low LD in Hanwoo compared to other beef cattle with high selection pressure for a long time.

### GWAS for Meat and Fat Color Traits

The GWAS for meat color identified 23 significant SNPs on BTA2, 14 and 24. Similar QTL regions have been reported in previous studies on similar traits (Reardon et al., 2010; Allais et al., 2014; Ribeca et al., 2014). This study was the first to examine GWAS for meat color in Hanwoo beef cattle; however, meat color has been studied in other beef cattle populations. For example in a Charolais population, a QTL located on BTA2 at 8.0 Mb reported by Allais et al. (2014). This QTL was close to the potential QTL region detected on BTA2 at 9.5Mb in the current study. Studies by Reardon et al. (2010) and Ribeca et al. (2014) revealed a potential QTL associated with meat color (yellowness or redness) on BTA2 spanning 59.3–65.1 Mb in Irish crossbred and Piedmontese cattle, respectively. This finding is in agreement with the current study result that was detected a QTL on BTA2 at 53.5 Mb for meat color trait. This potential QTL included one of the most significant SNPs identified in this study (p < 1×10^−8^). Other regions have also previously detected on chromosome 14 and 24. Recently, Allais et al. (2014) reported a potential QTL that has been linked with meat color (reflectivity) at 6.7 Mb on BTA24 in French beef cattle; however, the QTL region identified in the current study was located at 53.9 Mb on the BTA24. Furthermore, the potential QTL identified on chromosome 14 has not been previously associated with meat color traits.

Seven candidate genes for meat color have been identified across the three chromosomes (BTA2, 14 and 24). Among those candidate genes, ENPP2 gene was located near to the most significant SNP (rs137372673) located on BTA14, and this SNP showed high LD (0.95) with the adjacent SNPs (**Figure 4A**). According to Morales et al. (2017), *ENPP2* gene was responsible for lipid degradation and metabolism in human muscle. Narita et al. (1994) revealed that the *ENPP2* gene was also associated with pigmentation in the cell. Furthermore, *FAM171B* gene was located near to the most significant SNP (rs210985952) on BTA2 that was associated with meat color, however, the function of this gene is not well known in livestock. Similarly, *POLI* was a candidate gene found on BTA24 near to the most significant SNP (rs380354915) that was associated with meat color and it has been shown to be involved in DNA replication in human, although it has not been explicitly linked to meat or cell color (Gordon and Campbell, 1991).

The QQ plot for fat color (**Figure 1**) illustrated that some potential bias may still exist and therefore the more stringent Bonferroni threshold was used to identify significant peaks. In addition to this, the GWAS result for fat color was smoothed with a running median of the *p*-values that spanned five adjacent SNPs to pinpoint the most significant peaks (**Supplementary Material Figure 1B**). Therefore, the most significant QTL regions associated with fat color were located on chromosome 7 and 21, with other potential regions on many other chromosomes. No potential QTL has been previously reported on BTA7 and 12 that are associated with fat color. However, more than 18 QTLs that are associated with fat color in beef cattle have been reported previously (Yuan and Xu, 2011; Han et al., 2012; Tian et al., 2013; Xia et al., 2016). Potential overlap for regions on BTA13 and 14 may exist for peaks that did not reach the Bonferroni threshold, and therefore, these regions may be worthy of further examination in future studies.

The candidate gene (*CPAMD8*) for fat color was found close to the most significant SNP on BTA 7. It has no prior GWAS evidence of association with fat color, but it has been linked to the fat composition in Human. Other genes, *CPAMD8* and *RHCG* have been linked to various processes involved in lipid cell structure and development but have not been previously associated with fat color (Li et al., 2004; Fouillen et al., 2018). Furthermore, *RHCG* was a nearby gene to those significant SNPs associated with fat color on BTA21 and the gene involved in ammonium transmembrane transport in the cell (Biver et al., 2008).

Imputed WGS data, as used in this study, allowed for the fine mapping of QTL regions. In contrast to studies using genotype densities such as 50K and HD, the identified QTL regions were more precise (narrow confidence interval of the genomic region) which helped in the identification of potential genes relating to the discussed traits. Some variation between our results and those previously described could also be due to the choice of significance level/p-value threshold used to identify significant SNPs. Some conjecture still remains regarding definitions of significance. In this study, we have used a combination of both an alternative (p < 1×10^−6^) and Bonferroni threshold along with downstream gene analysis to identify regions that are likely to be linked to QTL for the traits discussed. As stated previously, many researchers advocate alternative thresholds under appropriate circumstances. The Bonferroni threshold has been widely used for traditional GWAS; however, this threshold may no longer be appropriate for methods that utilise the mixed linear model (MLM) procedures as done in GCTA. The Bonferroni threshold would likely increases false negatives and therefore discards significant observations in the MLM model (Zhang et al., 2010; Bolormaa et al., 2011; Sun et al., 2013).

In the current study, we identified new QTL regions that were associated with meat quality traits across the genome that are not reported elsewhere. Many of the regions were located close to genes that were involved in biological processes that link closely to each of the traits discussed. Some of the identified QTL regions in the current study may be false positive results due to the relatively small sample size used in the study. More data is currently being collected for this population, and further validation of associations that were very close to the significance thresholds may be warranted in the future.

## Conclusions

This study has estimated genetic parameters for meat quality traits in Korean indigenous beef cattle (brown Hanwoo) using imputed WGS data. It provides valuable estimates of genetic parameters and key insight into regions that underpin variation for the traits discussed. In this study, 107 significant SNPs that were highly associated with meat quality traits were identified across 14 chromosomes. These regions were in close proximity to the genes *SFT2D3* (marbling) *and ENPP2* (meat color). The significant regions identified in this study may provide valuable biological information to improve genomic selection accuracy in Hanwoo beef cattle breeding programs.

## Data Availability Statement

The datasets in this manuscript are available at the National Agriculture of Biotechnology Information Center (NABIC) (accession number NV-0544-000001).

## Ethics Statement

The animal study was reviewed and approved by the Animal Care and Use Committee (NIAS) and the ethics committee approval number was 2015-150.

## Author Contributions

MB, CG, SC and JW conceived and designed the study. MB performed data analysis (GWAS) and drafted the manuscript. SC, ND and JW had a contribution in data analysis and imaging. SC, JW, ND and DL were responsible for editing the manuscript. CG was responsible for imputation of 50K and 777K genotype data to sequence level. DL, BP, MP and RH were responsible for phenotypic data collection, genotyping and in quality control of sequence data. SC was responsible for overall supervision task (responsibility for the research activity planning and execution, including mentorship and communicating all authors). All authors read and agreed on the contents of the manuscript.

## Conflict of Interest

The authors declare that the research was conducted in the absence of any commercial or financial relationships that could be construed as a potential conflict of interest.
