# Supplementary material for: Genome-Wide Association Study of Meat Quality Traits in Hanwoo Beef Cattle Using Imputed Whole-Genome Sequence Data
Source: Front Genet. 2019 Nov 29;10:1235. doi: 10.3389/fgene.2019.01235 (PMC6895209; doi:10.3389/fgene.2019.01235)
Supplement: Supplementary file 1 [file Image_1.pdf]

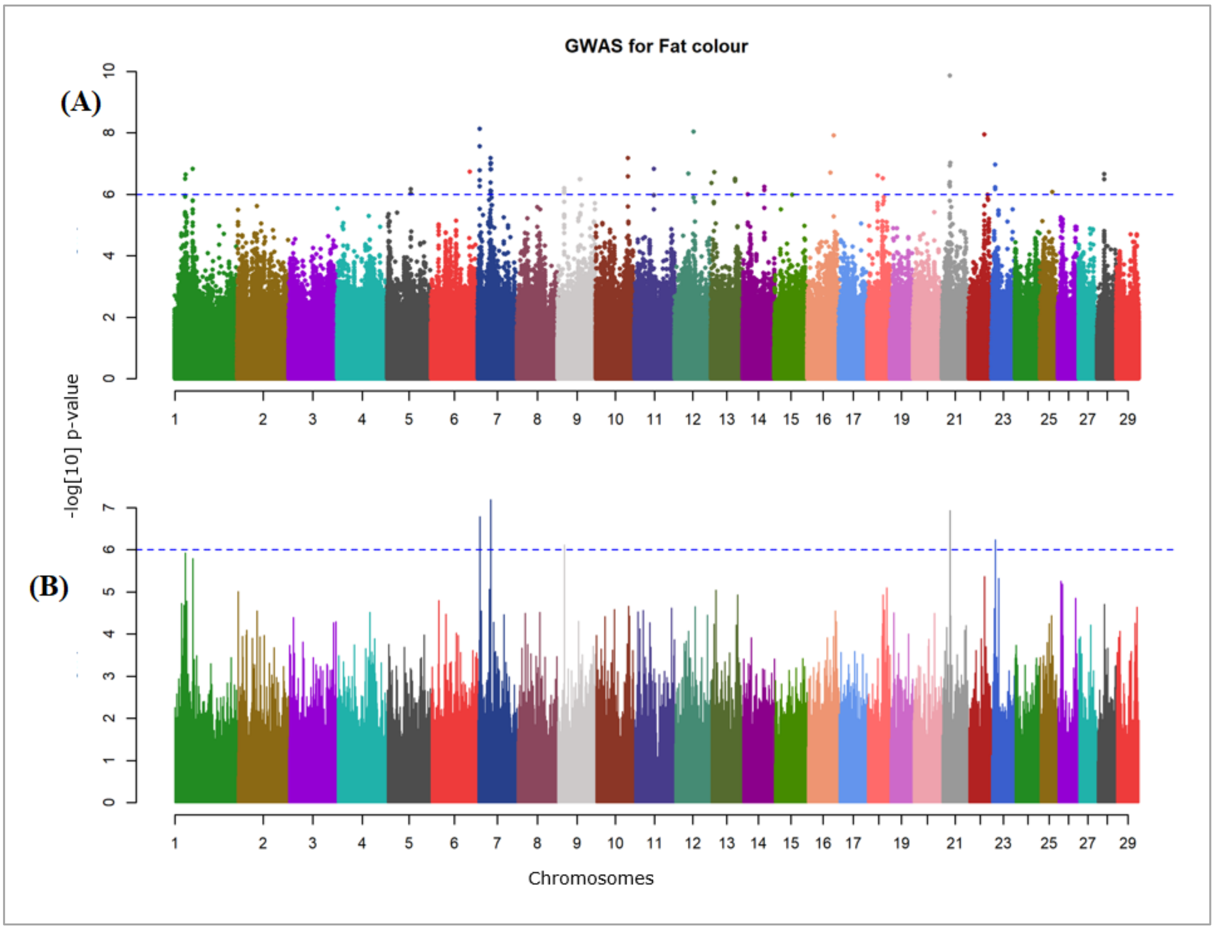

Supplementary Figure 1. Smoothed Manhattan plot for Fat colour trait. Manhattan plots of GWAS for Fat colour in Hanwoo beef cattle. The top panel was individual SNP result, and lower panel was smoothed median *P*-values using five SNP intervals.
