# Supplementary material for: Genome-Wide Association Study of Meat Quality Traits in Hanwoo Beef Cattle Using Imputed Whole-Genome Sequence Data
Source: Front Genet. 2019 Nov 29;10:1235. doi: 10.3389/fgene.2019.01235 (PMC6895209; doi:10.3389/fgene.2019.01235)
Supplement: Supplementary file 2 [file Table_1.docx]

**Supplementary material Table 1.** Chromosomes, significant SNPs and candidate genes associated with the studied meat quality traits. The table showed total significant SNPs per chromosome and top significant SNPs associated with meat quality traits (Marbling score, texture, meat and fat colour traits in 2110 Hanwoo bulls. Beta (β) is a substitution effect that explained by top significant SNP. The annotation column showed the type of genes associated with significant SNPs.

| Trait | Chromosome | Total significant  SNPs | Top SNPs | Genomic  position | Common  Allele | Minor  Allele | MAF | Effect size (β) | P-value | Annotation | Adjacent gene |
| --- | --- | --- | --- | --- | --- | --- | --- | --- | --- | --- | --- |
| Marbling score | 2 | 5 | rs43287038 | 4758180 | A | G | 0.017 | 1.107 | 1.06E-07 | Intergenic variant | *SFT2D3 and LIMS2* |
|  |  |  | rs136995876 | 4760585 | C | T | 0.017 | 1.107 | 1.06E-07 | Intergenic variant | *SFT2D3 and LIMS2* |
|  |  |  | rs132648300 | 4761900 | G | T | 0.017 | 1.107 | 1.06E-07 | Intergenic variant | *SFT2D3 and LIMS2* |
|  |  |  | rs136993172 | 4782843 | C | A | 0.017 | 1.039 | 1.16E-07 | Intron variant | *SFT2D3 and LIMS2* |
|  |  |  | rs134082813 | 53507272 | T | C | 0.153 | 0.156 | 1.3x10^-7^ | Intron variant | *ARHGAP15* |
|  | 12 | 4 | rs210129449 | 23375305 | T | C | 0.037 | 0.787 | 3.64E-07 | Intron variant | *FREM2* |
|  |  |  | rs208265955 | 23375323 | A | G | 0.037 | 0.787 | 3.64E-07 | Intron variant | *FREM2* |
|  |  |  | rs208621284 | 23378178 | G | A | 0.045 | 0.721 | 1.17E-07 | Intron variant | *FREM2* |
|  |  |  | rs210266948 | 23378344 | T | C | 0.037 | 0.782 | 6.45E-07 | Intron variant | *FREM2* |
|  | 16 | 10 | rs133022670 | 78561769 | G | C | 0.205 | 0.355 | 2.84E-07 | Intron variant | [*bta-mir-2284n*](http://asia.ensembl.org/bos_taurus/Gene/Summary?db=core;g=ENSBTAG00000044926) |
|  |  |  | rs137305161 | 78558219 | A | G | 0.205 | 0.355 | 2.99E-07 | Intron variant | [*bta-mir-2284n*](http://asia.ensembl.org/bos_taurus/Gene/Summary?db=core;g=ENSBTAG00000044926) |
|  |  |  | rs133850811 | 78558878 | G | C | 0.205 | 0.354 | 2.99E-07 | Intron variant | [*bta-mir-2284n*](http://asia.ensembl.org/bos_taurus/Gene/Summary?db=core;g=ENSBTAG00000044926) |
|  |  |  | rs136312829 | 78557763 | A | G | 0.205 | 0.353 | 3.64E-07 | Intron variant | [*bta-mir-2284n*](http://asia.ensembl.org/bos_taurus/Gene/Summary?db=core;g=ENSBTAG00000044926) |
|  | 24 | 12 | rs43760217 | 1983915 | T | A | 0.176 | 0.298 | 7.34E-06 | Intergenic variant | *-- and GALR1* |
|  |  |  | rs209257241 | 2251894 | G | A | 0.029 | 0.761 | 5.09E-07 | Intergenic variant | *-- and GALR1* |
|  |  |  | rs384383608 | 2287666 | C | T | 0.032 | 0.709 | 9.12E-07 | Intergenic variant | *-- and GALR1* |
|  |  |  | rs134591476 | 2300986 | C | T | 0.090 | 0.481 | 2.64E-07 | Intergenic variant | *-- and GALR1* |
|  |  |  | rs211167973 | 2302387 | C | G | 0.029 | 0.761 | 5.09E-07 | Intergenic variant | *-- and GALR1* |
| Texture | 12 | 4 | rs210129449 | 23375305 | T | C | 0.037 | -0.236 | 4.29x10^-7^ | Intron variant | *FREM2* |
|  |  |  | rs208265955 | 23375323 | A | G | 0.037 | -0.230 | 5.83x10^-7^ | Intron variant | *FREM2* |
|  |  |  | rs208621284 | 23378178 | G | A | 0.045 | -0.205 | 5.51x10^-7^ | Intron variant | *FREM2* |
|  |  |  | rs210266948 | 23378344 | T | C | 0.037 | -0.236 | 4.29x10^-7^ | Intron variant | *FREM2* |
|  | 29 | 9 | 29:19924266 | 19924266 | - | - | 0.042 | -0.255 | 8.14x10^-8^ | - | *ENSBTAT00000048899* |
|  |  |  | rs42766303 | 20061091 | C | T | 0.058 | -0.194 | 1.66x10^-6^ | Intergenic variant | *-- and LUZP2* |
|  |  |  | rs380974628 | 22807468 | G | C | 0.168 | -0.115 | 1.94x10^-6^ | Intron variant | *ANO5* |
| Meat colour | 2 | 9 | rs210985952 | 9570714 | A | T | 0.019 | 0.328 | 8.14x10^-7^ | Intron variant | *FAM171B* |
|  |  |  | rs207766300 | 9573053 | G | A | 0.019 | 0.328 | 8.14x10^-7^ | Intron variant | *FAM171B* |
|  |  |  | rs209498005 | 9576509 | C | T | 0.019 | 0.328 | 8.14x10^-7^ | Intron variant | *FAM171B* |
|  |  |  | rs110677532 | 9576733 | T | C | 0.021 | 0.281 | 8.60x10^-6^ | Intron variant | *FAM171B* |
|  | 14 | 8 | rs110820924 | 82246035 | T | C | 0.264 | -0.105 | 3.18x10^-6^ | Intergenic variant | *--and SNX16* |
|  |  |  | rs382546607 | 82249097 | A | C | 0.271 | -0.115 | 2.57x10^-7^ | Intergenic variant | *--and SNX16* |
|  |  |  | rs134557330 | 83349444 | T | C | 0.146 | 0.121 | 4.77x10^-6^ | Intergenic variant | *SLC10A5 and ENPP2* |
|  |  |  | rs137372673 | 83349464 | C | G | 0.089 | 0.174 | 1.63x10^-7^ | Intergenic variant | *SLC10A5 and ENPP2* |
|  |  |  | rs133028626 | 83349474 | T | C | 0.093 | 0.155 | 2.05x10^-6^ | Intergenic variant | *SLC10A5 and ENPP2* |
|  | 24 | 5 | rs110365059 | 51436913 | G | A | 0.273 | -0.109 | 8.50x10^-7^ | Intergenic variant | *CXXC1 and SKA1* |
|  |  |  | rs208112592 | 51469591 | A | G | 0.280 | -0.107 | 1.26x10^-6^ | Intergenic variant | *ENSBTAG00000034555* |
|  |  |  | rs380354915 | 53956767 | C | T | 0.035 | 0.225 | 6.25x10^-6^ | Intergenic variant | *-- and POLI* |
|  |  |  | rs382619136 | 53960102 | C | T | 0.035 | 0.225 | 6.25x10^-6^ | Intergenic variant | *-- and POLI* |
|  |  |  | rs381164398 | 53962726 | T | C | 0.035 | 0.225 | 6.25x10^-6^ | Intergenic variant | *-- and POLI* |
| Fat colour | 7 | 10 | rs210647091 | 38135632 | G | T | 0.028 | -0.11 | 7.45x10^-9^ | Intron variant | *CPAMD8* |
|  |  |  | rs382836662 | 37837021 | G | A | 0.012 | -0.17 | 6.60x10^-8^ | Intergenic variant | *CPAMD8 and SIN3B* |
|  |  |  | rs446548113 | 3813563 | G | A | 0.012 | -0.17 | 9.89x10^-8^ | Intergenic variant | *-- and RF00619* |
|  | 10 | 3 | rs43646756 | 89619111 | C | T | 0.016 | -0.14 | 6.74x10^-8^ | Downstream gene variant | *TMED8* |
|  |  |  | rs43644178 | 89643352 | A | T | 0.020 | -0.12 | 2.62x10^-7^ | Intron variant | *TMED8* |
|  |  |  | rs43644179 | 89645544 | C | A | 0.016 | -0.14 | 6.74x10^-8^ | Intron variant | *TMED8* |
|  | 12 | 2 | rs456360935 | 29879355 | A | T | 0.014 | 0.15 | 2.10x10^-7^ | Intron variant | *ENSBTAG00000016052* |
|  |  |  | 12:46590012 | 46590012 | - | - | 0.056 | -0.08 | 9.17x10^-9^ | - | *No adjacent genes* |
|  | 16 | 4 | 16:69913004 | 69913004 | - | - | 0.171 | -0.05 | 1.24x10^-8^ | - | *No adjacent genes* |
|  |  |  | 16:61322829 | 61322829 | - | - | 0.055 | -0.08 | 2.01x10^-7^ | - | *No adjacent genes* |
|  |  |  | rs383699790 | 61324080 | T | C | 0.055 | -0.08 | 2.01x10^-7^ | Intergenic variant | *RASAL2 and TEX35* |
|  |  |  | rs385877027 | 61327931 | G | C | 0.055 | -0.08 | 2.01x10^-7^ | Intergenic variant | *RASAL2 and TEX35* |
|  |  |  | rs465417325 | 61328149 | C | T | 0.055 | -0.08 | 2.01x10^-7^ | Intergenic variant | *RASAL2 and TEX35* |
|  | 21 | 22 | rs385466765 | 21384712 | C | G | 0.011 | -0.17 | 1.18x10^-7^ | Upstream gene variant | *RHCG* |
|  |  |  | rs135276961 | 21384807 | T | C | 0.011 | -0.17 | 1.18x10^-7^ | Upstream gene variant | *RHCG* |
|  |  |  | rs456186882 | 21384841 | G | A | 0.011 | -0.17 | 1.18x10^-7^ | Upstream gene variant | *RHCG* |
|  |  |  | rs210519496 | 21385057 | G | A | 0.011 | -0.17 | 1.18x10^-7^ | Upstream gene variant | *RHCG* |
|  |  |  | rs476169388 | 21385184 | G | T | 0.011 | -0.17 | 1.18x10^-7^ | Upstream gene variant | *RHCG* |
